# Prevalence and Risk Factors of Urolithiasis Among the Population of Hail, Saudi Arabia

**DOI:** 10.7759/cureus.26983

**Published:** 2022-07-18

**Authors:** Akram A Bokhari, Hadi A Aldarwish, Saleh A Alsanea, Mohammed A Al-Tufaif, Sulaiman A Alghaslan, Ali A Alghassab, Basil B Alshammari, Ali A Al-Tufaif

**Affiliations:** 1 Urology, University of Hail College of Medicine, Hail, SAU; 2 Medicine and Surgery, University of Hail College of Medicine, Hail, SAU

**Keywords:** saudi arabia, risk factors, nephrolithiasis, renal stones, urolithiasis

## Abstract

Background

Urolithiasis is the formation of calculi in the urinary system. It is a public health concern worldwide that can lead to serious long-term consequences. Age, gender, dietary habits, and physical activity levels are all factors that increase the risk of urolithiasis formation. Furthermore, the presence of comorbid medical conditions such as diabetes and hypertension are other major risk factors. Among the most prominent determinants that raise the likelihood of acquiring urolithiasis is exposure to high temperatures, especially in middle-aged men. Consequently, Saudi residents are two and a half times more prone than the global average to develop urolithiasis, especially those in the Kingdom’s hottest regions.

Methodology

This cross-sectional study assessed the self-reported prevalence and non-nutritional risk factors of urolithiasis among the population of Hail, Saudi Arabia, through an electronic questionnaire. The questionnaire contained 16 questions divided into three categories. Participants’ permission was obtained before completing the questionnaire. The Statistical Package for Social Sciences (SPSS) version 22 (IBM Corp., Armonk, NY, USA) was used to analyze the data.

Results

Of the 1150 participants with a mean age of 26.3 ± 12.8 years old, nearly half were males (50.9%). Urolithiasis was detected among 158 (13.7%) participants. The following factors showed significant relation with having urolithiasis: increased age, male gender, a low level of education, diabetes, hypertension, and hyperthyroidism. A family history of renal stones was also associated with double the risk of having urolithiasis.

Conclusion

The results showed a high prevalence of urolithiasis in the Hail region, with many risk factors associated with it. It is important to support and promote awareness campaigns that address the critical risk factors of urolithiasis. Further studies should be conducted to arrive at a better understanding of the association between non-nutritional risk factors and developing urolithiasis.

## Introduction

Urolithiasis refers to the formation of urinary calculi in the urinary system [1]. Age, gender, ethnic groupings, local climate, dietary habits, physical activity, and occupation are all risk factors that can contribute to the development of urolithiasis [2]. The presence of comorbid medical conditions such as diabetes, hypertension, and obesity are other major factors [3]. The overall probabilities of forming stones vary from country to country; nevertheless, it is a public health concern worldwide [4].

According to recent studies, kidney stones are more than an acute event, since they can lead to serious long-term consequences. Therefore, efforts should be made to reduce the burden of kidney stones [5]. Additionally, kidney stones can also increase healthcare costs. Previous studies have shown different international efforts in the form of comparing the incidence of urolithiasis in different population groups to define risk factors for urolithiasis. [6]. These valuable investigations primarily propose an interventional trial or a research question to examine if the defined risk factors can cause kidney stones and if managing these risk factors can help prevent nephrolithiasis from spreading [7]. Consequently, the quality of life for the general population is improved [8].

Stone formation is a multifactorial disease, involving environmental and metabolic aspects. Exposure to high temperatures is one of the most prominent determinants that raises the likelihood of acquiring stones, especially in men between the ages of 30 and 60 [9]. Due to the region’s climate, Saudis are two and a half times more susceptible than others to develop urolithiasis, especially those in the Kingdom’s hottest regions [10]. An appropriate detection method using well-defined samples from a population is important for accurately assessing the effects of urolithiasis rate fluctuations and their possible risk factors in the population of Hail, Saudi Arabia. This study was thus undertaken to determine the prevalence and non-nutritional risk factors of urolithiasis in Hail.

## Materials and methods

Research design and setting

This cross-sectional study was approved by the University of Hail’s Research Ethics Committee (approval no. H-2022-164). The study was conducted between April and June 2022. It aimed to estimate the prevalence and non-nutritional risk factors of urolithiasis among the Hail population, through an electronic questionnaire written in Arabic and distributed via multiple social media applications (though mainly Twitter and WhatsApp). Information was kept private per Google’s privacy policies. The study was conducted in accordance with the Helsinki Declaration’s principle regarding studies involving human participants.

Sample size

The equation that was adopted in the measurement of sample size is ss = (z2×p×q)/d2. Where ss = sample size, z = 1.96, p = 0.5, q = (1-p) = 0.5, and d = sampling error at 3%. As stated by this equation, the lowest acceptable sample size for establishing a study with ± 3% error and 95% confidence interval (CI) is 1066. However, we added a margin of error and increased the sample size to 1150. The criteria included in this study encompassed those who are 18 years or older, live in the Hail region, and are willing to participate in this study. We excluded participants who live outside Hail, are younger than 18 years old, or who made incomplete submissions.

Development and application of the questionnaire

The research team created a questionnaire to estimate the prevalence and non-nutritional risk factors of urolithiasis. The questionnaire contained 16 questions divided into three categories. The first category gathered demographic information from participants, the second concerning past medical history, and the third dealt with the self-reported prevalence of urolithiasis. Participants’ permission was obeforeprior to completing the questionnaire. The questionnaire was translated from English to Arabic by a translator.

Statistical analysis

After data were extracted, it was revised, coded, and fed into the statistical software Statistical Package for Social Sciences (SPSS) version 22 (IBM Corp., Armonk, NY, USA). All statistical analysis was done using two-tailed tests. A p-value less than 0.05 was statistically significant. We used WHO classification of body mass index (BMI) to classify participants as normal, overweight, and obese. Descriptive analysis based on frequency and percent distribution was conducted for all variables, including participants’ personal data, education level, and medical history. Crosstabulation was used to assess factors associated with urolithiasis, including participants’ personal data and medical history. Relations were tested using the Pearson chi-square test and exact probability test for small frequency distributions. Adjusted binary logistic regression was used, including whole factors related to renal stone (urolithiasis) formation. The forced entry model was also used, whereby all independent variables were tested in one block to assess their association while controlling for the effects of other variables in the model. Multicollinearity was checked by running collinearity in the multiple linear logistic regression and was measured by a tolerance < 0.10 and a variance inflation factor (VIF) > 10. There was no evidence of collinearity in the adjusted model. The statistical significance level was set at P < 0.05. The adjusted exponentiation of the B coefficient-Exp (B) value or odds ratios (OR) and 95% confidence interval (CI) were displayed for each included variable.

## Results

A total of 1150 participants completed the study questionnaire after a pilot study was conducted first to statistically determine the reliability of the survey using Cronbach alpha, it was found reliable with >0.60. Participants ages ranged from 18 to 68 years with a mean age of 26.3 ± 12.8 years old. Of the total participants, 585 (50.9%) were males and 1100 (95.7%) were Saudi. As for education, 833 (72.4%) had a university level of education/above while 302 (26.350 had mid-level education/secondary. A total of 268 (23.3%) of the study participants were health care workers (HCWs) and 464 (40.3%) had normal weight while 379 (33%) had overweight, and 307 (26.7%) were obese (Table 1).

**Table 1:** Bio-demographic data of study participants from Hail, Saudi Arabia HCW: Health care worker, BMI: Body mass index

Bio-demographic data	Number of participants	%
Age in years		
18-25	487	42.3%
26-35	212	18.4%
36-50	317	27.6%
51-60	106	9.2%
> 60	28	2.4%
Gender		
Male	585	50.9%
Female	565	49.1%
Nationality		
Saudi	1100	95.7%
Non-Saudi	50	4.3%
Education		
Primary/below	15	1.3%
Middle/secondary	302	26.3%
University/above	833	72.4%
Job title		
HCW	268	23.3%
Others	882	76.7%
BMI		
Normal	464	40.3%
Overweight	379	33.0%
Obese	307	26.7%

One hundred and eighty-three (15.9%) participants were smokers, 124 (10.8%) were diabetic, 121 (10.5%) were hypertensive, 33 (2.9%) complained of intestinal disease, 28 (2.4%) had gout, 29 (2.5%) complained of hypothyroidism, eight (0.7%) had chronic kidney disease. Of the participants, 507 (44.1%) had a family history of urolithiasis, 158 (13.7%) complained of urolithiasis, while 992 (86.3%) did not (Table 2).

**Table 2:** Medical history of study participants of Hail, Saudi Arabia DM: Diabetes mellitus, HTN: Hypertension

Medical history	Number of participants	%
Smoker		
Yes	183	15.9%
No	967	84.1%
Chronic diseases		
None	829	72.1%
DM	124	10.8%
HTN	121	10.5%
Hyperthyroidism	28	2.4%
Intestinal disease	33	2.9%
Chronic kidney disease	8	.7%
Gout	28	2.4%
Hypothyroidism	29	2.5%
Others	42	3.7%
Family history of urolithiasis		
Yes	507	44.1%
No	643	55.9%
Had urolithiasis		
Yes	158	13.7%
No	992	86.3%

Of the total participants, 27.4% aged 51-60 years had renal stones versus 6.4% of those aged 18 to 25 years, with a recorded statistical significance (P = 0.001). Renal stones were reported among 17.3% of male participants compared with 10.1% of females (P = 0.001). And 18.2% of participants with middle/secondary education level complained of urolithiasis in comparison with 13.3% of others with a low level of education (P = 0.031). Additionally, 14.9% of those who were not HCWs complained of urolithiasis compared with 10.1% of HCWs (P = 0.047) (Table 3).

**Table 3:** Prevalence of urolithiasis as per the socio-demographic data of participants from Hail, Saudi Arabia P: Pearson X2 test, $: Exact probability test * P < 0.05 (significant)

Socio-demographic data	Had urolithiasis				P-value
	Yes		No		
	No	%	No	%	
Age in years					0.001*^$^
18-25	31	6.4%	456	93.6%	
26-35	38	17.9%	174	82.1%	
36-50	53	16.7%	264	83.3%	
51-60	29	27.4%	77	72.6%	
> 60	7	25.0%	21	75.0%	
Gender					0.001*
Male	101	17.3%	484	82.7%	
Female	57	10.1%	508	89.9%	
Nationality					0.956
Saudi	151	13.7%	949	86.3%	
Non-Saudi	7	14.0%	43	86.0%	
Education					0.031*
Primary/below	2	13.3%	13	86.7%	
Middle/secondary	55	18.2%	247	81.8%	
University/above	101	12.1%	732	87.9%	
Job title					0.047*
HCW	27	10.1%	241	89.9%	
Others	131	14.9%	751	85.1%	
BMI					0.131
Normal	55	11.9%	409	88.1%	
Overweight	51	13.5%	328	86.5%	
Obese	52	16.9%	255	83.1%	

Of the participants, 20.8% who were smokers complained of urolithiasis, compared with 12.4% of non-smokers (P = 0.003). Furthermore, 29% of diabetic participants had urolithiasis compared with 11.9% of non-diabetic participants (P = 0.001). Additionally, urolithiasis was reported among 28.9% of hypertensive participants in comparison with 12% of normotensive participants (P = 0.001). Around 32.1% of participants with hyperthyroidism complained of urolithiasis versus 13.3% of others (P = 0.004). Urolithiasis was detected among 42.9% of participants with gout compared with 13% of those without (P = 0.001). Moreover, 17.6% of participants with a family history of renal stones had urolithiasis versus 10.7% of others who had no such history (P = 0.001) (Table 4).

**Table 4:** Prevalence of urolithiasis by the medical and past history of participants from Hail, Saudi Arabia P: Pearson X2 test, $: Exact probability test, HTN: Hypertension, DM: Diabetes mellitus * P < 0.05 (significant)

Past medical history	Had urolithiasis				P-value
	Yes		No		
	No	%	No	%	
Smoker					0.003*
Yes	38	20.8%	145	79.2%	
No	120	12.4%	847	87.6%	
DM					0.001*
Yes	36	29.0%	88	71.0%	
No	122	11.9%	904	88.1%	
HTN					0.001*
Yes	35	28.9%	86	71.1%	
No	123	12.0%	906	88.0%	
Chronic kidney disease					-
Yes	4	50.0%	4	50.0%	
No	0	0.0%	0	0.0%	
Intestinal disease					0.206^$^
Yes	7	21.2%	26	78.8%	
No	151	13.5%	966	86.5%	
Hypothyroidism					0.103^$^
Yes	1	3.4%	28	96.6%	
No	157	14.0%	964	86.0%	
Hyperthyroidism					0.004*
Yes	9	32.1%	19	67.9%	
No	149	13.3%	973	86.7%	
Gout					0.001*
Yes	12	42.9%	16	57.1%	
No	146	13.0%	976	87.0%	
Others					0.725
Yes	5	11.9%	37	88.1%	
No	153	13.8%	955	86.2%	
Family history of urolithiasis					0.001*
Yes	89	17.6%	418	82.4%	
No	69	10.7%	574	89.3%	

Among all included factors, the following showed significant relation with having urolithiasis. First, increased age was associated with a 40% greater likelihood of having renal stones (OR = 1.40; 95% CI: 1.19-1.66) when keeping all other factors constant. Furthermore, male participants had a 59% greater likelihood of having renal stones than females (OR = 1.59; 95% CI: 1.06-2.37). Participants with a low level of education showed a significantly higher likelihood of urolithiasis than the university-educated group by 78% (OR = 1.78; 95% CI: 1.01-8.84). Additionally, diabetic participants had an 82% greater likelihood of urolithiasis (OR = 1.82; 95% CI: 1.10-3.03). Hypertensive participants showed a 56% greater likelihood of urolithiasis than normotensive (OR = 1.56; 95% CI: 1.11-3.69). Hyperthyroidism was associated with more than double the risk of having urolithiasis (OR = 2.38; 95% CI: 1.13-5.65). Last, a family history of renal stones also doubled the risk of having urolithiasis (OR = 1.99; 95% CI: 1.39-2.85) (Table 5).

**Table 5:** Multiple logistic regression for predictors of urolithiasis among study participants, Hail, Saudi Arabia OR a: Adjusted odds ratio, CI: Confidence interval, DM: Diabetes mellitus, HTN: Hypertension, FH: Familial hypercholesterolemia * P < 0.05 (significant)

Factor	P-value	OR_a_	95% CI	
			Lower	Upper
Age in years	0.001*	1.40	1.19	1.66
Male gender	0.024*	1.59	1.06	2.37
Primary/below	0.048*	1.78	1.01	8.84
DM	0.020*	1.82	1.10	3.03
HTN	0.043*	1.56	1.11	3.69
Hyperthyroidism	0.047*	2.38	1.13	5.65
FH	0.001*	1.99	1.39	2.85

## Discussion

Urolithiasis is one of the most prevalent urinary tract disorders [11]. It is a multifactorial problem that can be affected by age, gender, diet, weather, and body mass index [12]. This study was therefore conducted to assess the prevalence and non-nutritional risk factors of urolithiasis in the Hail region of Saudi Arabia. Of 1150 participants, 158 (13.7%) had urolithiasis; 992 (86.3%) did not. Our study showed a high prevalence of urolithiasis among Hail population, in comparison with another study conducted by Safdar et al. [10]. However, the global prevalence rate is 4% to 20% [13]. Data analysis identified several adjusted determinants for developing urolithiasis. These were: older age, male, smoker, comorbidity, family history, and non-health care worker.

In this study, the highest prevalence of urolithiasis was among those between 51 to 60 years old. The prevalence increased with age. These results are similar to other studies conducted by Baatiah et al. and Moudi et al. [14,15]. On the other hand, another study conducted in Jeddah and Riyadh shows a high prevalence of urolithiasis among participants aged between 18 to 30 years (33.70%) [16]. Furthermore, male participants were more prone to develop urolithiasis compared to female participants (OR = 1.59; 95% CI: 1.06-2.37). This finding was also observed by Anmar et al. and Scales et al. [17,18]. Moreover, a retrospective study in the Eastern region of Saudi Arabia shows a higher prevalence of nephrolithiasis among males (74.5%), with calcium oxalate being the most prevalent type [19]. However, this gender gap may be because of the protective effects of estrogen. Such an explanation would be better supported by the finding that this gender gap declines in post-menopausal women [20].

In this study, we found that the relative risk of having urolithiasis is less in HCWs than non-HCWs. This is supported by another study done by Bos et al., which reported that most HCW respondents knew appropriate precautions against recurrent urolithiasis [21]. Concerning BMI, in the study of Taylor et al., high BMI was associated with the formation of urolithiasis [22]. This finding is consistent with the claim that a larger body size may lead to increased urinary excretion of calcium oxalate, and uric acid, which in turn increases the risk of forming calcium-containing kidney stones [23]. However, no significant association between BMI and urolithiasis was found in this study.

Tamadon et al. suggest smoking may be an independent risk factor for urolithiasis, and it was 2.06 times more common in stone formers than in controls [24]. One possible explanation is that cigarette smoking may increase serum cadmium and decrease urinary flow in healthy subjects which induce urolithiasis [25]. Moreover, in this study we reported that 20.8% of participants who were smokers had urolithiasis. Our study recorded the prevalence of urolithiasis in diabetic patients to be higher than non-diabetic patients. Previous studies also support this [26]. The relation between diabetes and urolithiasis has been largely explained by the effect of insulin resistance on urine pH and renal handling of ammonium and calcium [27].

Furthermore, the present study also showed a positive association between hypertension, gout, hyperthyroidism, and an increased risk of urolithiasis. Other studies also concur [28,29]. The literature indicated that patients with hypertension may have abnormalities of renal calcium metabolism, which increases the risk of developing urolithiasis [30]. Not surprisingly, the risk of kidney stones is higher in those with a positive family history, which is similar to our result [14].

Limitations

Since the survey was distributed online, it can be subjected to sampling bias. The questionnaire was not validated. Moreover, we did not explore the role of occupation on the population and the sample size does not reflect the true population of the region. Also, the prevalence is self-reported which may not reflect the true prevalence of urolithiasis. Furthermore, a prospective observational study could be better at detecting any significant relationship between risk factors and the development of urolithiasis.

## Conclusions

The study participants show a high prevalence of urolithiasis in the Hail region. In addition, the most significant factors for urolithiasis included male gender, low level of education, family history of urolithiasis, and old age. Diabetes, hypertension, and hyperthyroidism also had a significant impact.

This study, therefore, provides a database to inform people about the possibility of developing urolithiasis based on their demographic data and past medical history. However, it is important to support and promote awareness campaigns that address the critical risk factors of urolithiasis. Further studies should be conducted to better understand the association between non-nutritional risk factors and developing urolithiasis.
